# Silent spread of *mcr-9* in ESBL-producing *Enterobacteriaceae clinical isolates*, Jimma, Ethiopia

**DOI:** 10.1371/journal.pone.0336440

**Published:** 2025-11-18

**Authors:** Tsegaye Sewunet, Mohammad Razavi, Chaitanya Tellapragada, Christian G. Giske

**Affiliations:** 1 Division of Clinical Microbiology, Department of Laboratory Medicine, Karolinska Institutet, Stockholm, Sweden; 2 Department of Clinical Microbiology, Karolinska University Hospital, Stockholm, Sweden; The University of Texas Medical Branch at Galveston, UNITED STATES OF AMERICA

## Abstract

**Background:**

Mobile colistin resistance (MCR) genes, have been increasingly identified worldwide, but their presence and characteristics in Africa remain poorly understood. Herein, we characterized a silent *mcr-9* gene carried in *Enterobacteriaceae* from Ethiopia.

**Methods:**

In a study investigating genomic epidemiology of ESBL-producing Gram-negative bacilli from clinical samples in 2016, eleven isolates were found to encode *mcr-9* genes. Whole genome sequencing, combining Illumina and MinION Nanopore technologies were performed for in-depth investigation of the genome and understand the genetic contexts of *mcr-9* and its associated plasmids.

**Result:**

The *mcr-9* genes were detected in *Enterobacter cloacae* (n = 8), *Escherichia coli* (n = 1), *Klebsiella pneumoniae* (n = 1), and *K. michiganensis* (n = 1). They were isolated from urine (54.5%, 6/11), wound secretions (27.3%, 3/11), and fecal samples (n = 18.2%, 2/11). In addition to *mcr-9*, all isolates encoded *bla*_CTX-M-15_, and *aac (6’)-Ib-cr* among several other resistance genes. These isolates were susceptible to colistin (MIC ≤ 0.5 mg/L). The *E. cloacae* strains belong to three different sequence types (ST): ST114 (n = 3), ST184 (n = 3), ST254 (n = 1), and *Enterobacter mori* ST2197 (n = 1)), whereas the *E. coli* and the *K. pneumoniae* strains belonged to ST410 and ST337. *IncHI2* and *IncHI2*A plasmid replicons were present in all isolates. Although the genetic content of plasmids carrying the *mcr-9* genes varied, the genetic contexts surrounding all *mcr* genes within ±10kb region were largely consistent, mostly flanked by composite transposons such as IS*26*, and IS*903B*.

**Conclusion:**

A silent *mcr-9* gene was detected among ESBL-producing *Enterobacteriaceae* isolates. The *IncHI2* plasmids encoding the *mcr-9* had 25% genetic content dissimilarity.

## Introduction

The global spread of antimicrobial resistance (AMR) is fueled by mobile genetic elements (MGEs) [1]. They are drivers of the plasticity of bacterial genomes through intercellular mobility of necessary functions such as antibiotic resistance genes (ARGs) between different species [2,3]. The important human pathogens, including *Enterobacteriaceae* and other Gram-negative bacilli, have recruited mobile ARGs conferring resistance to antibiotics with broad spectrum activity including colistin and carbapenems. These antimicrobials are designated as critically important, and alternative therapeutic agents are urgently needed against resistant strains [4], particularly in low- and -middle income settings where novel antimicrobials such as the new beta-lactam/beta-lactam inhibitor combinations are not yet available.

The main colistin resistance mechanism was historically known as chromosomal mutations. However, the discovery of plasmid-mediated colistin resistance (*mcr-1*) in 2015 highlighted the presence of a new mechanism with ample opportunity to spread among diverse bacterial species [5]. Since the first report of *mcr-1*, increased prevalence of *mcr*-genes, and about ten different variants of *mcr* were reported [6]. The *mcr-9* was identified in *Salmonella enterica* serovar Typhimurium [7], *E. coli* [8] and other species [6]. The *mcr* family has been reported from several sources including human, environmental, food, and animals [9,10]. Multiple studies detected *mcr*-genes from clinical samples, including bloodstream infections from countries such as Italy [11], Switzerland [12], China [13], Philippines [14], and Czeck Republic [15]. Understanding the transmission mechanisms and the spread of *mcr* genes could be important to contain their further dissemination.

In this study, plasmids from clinical isolates of *Enterobacteriaceae* and carrying *mcr-9* were characterized. The genetic context of *mcr-9*, the antibiotic susceptibility and resistome profiles, and genomic epidemiology of the bacterial hosts were studied. In Africa, data regarding *mcr-9* is limited and it is difficult to predict the situation. Also, when it occurs in low-income health care settings where colistin has not been used, or patients had no travel history, it requires more attention to understand the mechanism of transmission. In this regard, our study shed light on the co-selection and silent spread of the *mcr-9* gene among ESBL-producing *Enterobacteriaceae* which might later revert to resistance phenotype under selection pressure when colistin is used [16].

## Materials and methods

Ethical approval was given by the Ministry of Science and Technology of Ethiopia- National Ethics and Review committee (NERC) Ref No. 3–10/150/2016.

### Strain collection

Isolates carrying *mcr-9* were collected as part of genome-based cross-sectional survey of ESBL-producing *Enterobacteriaceae* at Jimma Medical Center, Ethiopia. As shown in our previous work, [17,18] we enrolled a total of 1,087 patients with suspected bacterial infection. Species identification was done using matrix assisted laser desorption ionization-time of flight mass spectrometry (MALDI-TOF) at the Department of Clinical Microbiology, Karolinska University Hospital, Solna. Antimicrobial susceptibility testing was done by disk diffusion using EUCAST guidelines (https://www.eucast.org/ast_of_bacteria) and broth microdilution for novel antibiotics tested against these isolates. Eleven ESBL-producing isolates were found to encode the *mcr-9* gene upon resistome analysis. The genetic context surrounding the *mcr*-genes as well as the plasmids carrying them were studied. These isolates, and their mutant variants were tested for susceptibility to increased concentration colistin.

DNA extraction and sequencing: Genomic DNA was extracted using EZ1^®^DNA Tissue Kit (QIAGEN) by using the EZ1 Advanced DNA Bacteria Card on EZ1 Advanced extraction system. NEXTRA-XT kits (Illumina) were used for library preparation and sequencing was performed on Illumina (HiSeq2500) platform at *Science for Life Laboratories*, Stockholm.

All the sequenced isolates were annotated as follows: First, the quality of the reads was checked using FastQC. Then, the paired-end reads with low-quality bases were trimmed and filtered using Trim-Galore tool [19] The remaining paired-end and single reads were assembled into longer contigs using SPAdes (version 3.13). Then, open reading frames (ORFs) were predicted by prodigal (version 2.6.3). For annotation, Diamond (version 2.0.4) were used to compare ORFs against the comprehensive antimicrobial resistance database (CARD, version 3.1), Virulence factor database (VFDB), Non-redundant NCBI protein database (downloaded Feb. 2022). The assembled draft genomes were also used to query bacterial genome analysis at Center for Genomic Epidemiology (https://cge.food.dtu.dk/services/MLST Last access: September 2023) to identify multi-locus sequence typing. Capsular and O-lipopolysaccharide typing were identified using Kaptive (https://kaptive-web.erc.monash.edu/ last access: September 2023).

Characterization of the plasmids: After identifying *mcr-9* in the characterized isolates, nine of them were selected for sequencing with Nanopore MinION technology. The resulting long reads enabled reconstructing of the genetic contexts around *mcr-9* by employing a hybrid assembly using Unicycler (version 0.4.8). The resulted drafted genomes were annotated using the previously mentioned pipeline. Plasmids were identified by mapping the contigs against the NCBI plasmid collection (downloaded Feb. 2022) using BLASTn algorithm in BLAST+ program [20]. The incompatibility groups were identified using the PlasmidFinder tool. Moreover, the content of the recovered plasmids was compared as follows: First, all the predicted ORFs on plasmids were clustered using CD-HIT tool (version 4.8.1) with 90% similarity. Then, a matrix was created, in which columns were different clusters of ORFs and rows were identified plasmids and values were the number of discovered clusters on plasmids. Using Bray-Curtis measure, the dissimilarity between plasmids were calculated. Finally, the dendrogram and PCoA plot were produced in R using the following packages: vegan (version 2.5), ggdendro (version 0.1.23), ggplot2 (version 3.3.5).

Moreover, the immediate (±5 kb) genetic context around *mcr-9* were analyzed using progressiveMauve (version 2.4.0). The reference genome was a region of plasmid discovered in *Salmonella enterica* that was harboring *mcr-9* and confer resistance to colistin (NCBI-accession: CP006057.1[11014:22633]). The visualization was preformed using an in-house Mauve-viewer program (https://github.com/xrazmo/mauve-viewer).

Antimicrobial susceptibility testing: Minimum inhibitory concentrations (MICs) were determined using a customized broth microdilution plate, MDROXF (sensititer plates from Thermo Fisher Scientific, Waltham, United States). The Sensititer plates panel contains the following antibiotics: amikacin, aztreonam, cefepime, colistin, imipenem, meropenem, piperacillin-tazobactam, tobramycin, tigecycline, ceftazidime-avibactam, meropenem, imipenem, eravacycline, ceftolozane-tazobactam, imipenem-relebactam, and meropenem-vaborbactam.

## Result

### Isolation of strains and patient characteristics

The *mcr-9* genes were detected in four different species of *Enterobacteriaceae,* all the isolates were ESBL-producing. The prevalence of *mcr-9* gene among ESBL-producing *Enterobacterales* was 3.5% (11/312), however, it varied with species, *E. cloacae* (12.9%, 8/62), *Klebsiella* spp. (*K. pneumoniae* and *K. michiganensis*) (1.8%, 2/109)), and *E. coli* (0.7%, 1/141). All strains were isolated from patients admitted to the hospital. Two of the strains (*K. pneumoniae,* and *E. coli)*, were isolated from patients admitted at pediatric ward. Similarly, two isolates of *E. cloacae* were from the medical ward, and the rest of *E. cloacae* (n = 6), and *K. michiganensis* (n = 1) were isolated from the surgical ward. About 64% (7/11) were isolated from patients admitted with urinary tract infections and most of these patients had underlying chronic disease. Details of the clinical information regarding types of infection, factors such as prior use of antibiotics, and types of underlying chronic diseases are presented in Table 1.

**Table 1:** Patients’ socio-demographic characteristics and clinical data.

Isolate ID	Age	Sex	Specimens	Current antibiotic use	^1^Suspected infection	Underlying chronic illness	MCR-9-positive
P004kp	3	M	Stool	ampicillin	Diarrhea	Malnutrition/SAM^**^	*K. pneumoniae*
P165e	10	M	Stool	No	Diarrhea	Malnutrition/SAM^**^	*E. coli*
S082km	24	M	Urine	ceftriaxone	Urinary tract infection	Urethral stricture	*K. michiganensis*
M016ECL	50	M	Urine	No	Urinary tract infection	Retroviral infection	*E: cloacae*
M481ECL	57	M	urine	ceftriaxone, metronidazole	Urinary tract infection	Hemiparesis	*E. cloacae*
S164ECL	50	F	Wound swab	Ceftriaxone	Wound infection	Breast cancer	*E. cloacae*
S202ECL	20	M	Urine	ampicillin, chloramphenicol	Urinary tract infection	Femoral fracture	*E. cloacae*
S249ECL	20	M	Wound swab	No	Wound infection	No	*E. cloacae*
S257ECL	70	M	Urine	No	Urinary tract infection	Benign prosthetic hyperplasia	*E. cloacae*
S284ECL	58	M	Urine	ceftriaxone	Urinary tract infection	Obstructive uropathy	*E. cloacae*
S304ECL	23	M	Urine	ceftriaxone, metronidazole	Urinary tract infection	Urethral stricture	*E. cloacae*

SAM^**^: Severe acute malnutrition.

### Minimum inhibitory concentration of isolates

In all isolates MICs in the susceptible range were observed for colistin, meropenem, imipenem, meropenem/vaborbactam, and imipenem/relebactam. Also, for tigecycline, a low MIC (<= 1 mg/L) was observed, suggesting no acquired resistance. In most of the isolates, MICs in the resistant range was observed for aztreonam 100% (11/11), tobramycin 63.6%, (7/11), piperacillin/tazobactam 63.6% (7/11), and cefepime (63.6%, 7/11). Details about antimicrobial susceptibility are presented in Table 2.

**Table 2:** Minimum inhibitory concentration of the mcr-9 isolates to commonly prescribed antibiotics.

Species ID		Antimicrobials and minimum inhibitory concentrations (MIC)														
		Amikacin	Aztreonam	Cefepime	Colistin	Fosfomycin	Imipenem	Meropenem	Piperacillin/ tazobactam	Tobramycin	Tigecycline	Ceftazidime/ avibactam	Eravacycline	t azobactam	Imipenem/ relebactam	Meropenem/ vaborbactam
p165e	*E. coli*	8	32	16	0.5	16	1	0.12	4	0.5	0.5	4	0.25	0.25	0.5	0,06
p004kp	*K. pneumoniae*	2	32	2	0.5	16	1	0.12	32	4	0.5	1	0.12	1	2	0.06
s082km	*K. michganensis*	32	32	16	0.5	16	1	0.12	4	0.5	0.5	0.5	0.25	0.25	0.5	0.06
m016ECL	*E. mori*	2	32	1	0.5	16	1	0.12	4	0.5	0.5	0.25	0.008	0.25	0.06	0.06
m481ECL	*E. cloacae*	>8	32	1	0.5	16	1	0.12	4	0.5	0.5	0.25	0.008	0.25	1	0.06
s164ECL	*E. cloacae*	4	32	4	0.5	16	1	0.12	32	4	0.5	0.25	0.008	0.25	0.06	0.06
s202ECL	*E. cloacae*	8	32	16	1	16	1	0.12	32	4	0.5	0.25	0.008	0.25	0.5	0.06
s249ECL	*E. cloacae*	8	32	16	0.5	16	1	0.25	32	4	0.5	4	0.5	8	0.5	0.06
s257ECL	*E cloacae*	8	32	16	0.5	16	1	0.25	32	4	0.5	2	>0.5	8	0.5	0.12
s284ECL	*E cloacae*	8	32	16	0.5	16	1	0.12	32	4	1	2	>0.50	8	0.25	0.06
s304ECL	*E. cloacae*	>8	32	16	1	16	1	0.12	32	4	0.5	0.25	0.12	0.25	0.5	0.06

Key: Resistant Susceptible.

### Molecular characteristics of strains

Table 3 shows that each of the isolates carried at least 9–14 different ARGs in addition to *mcr-9*. Notably, they harbored ESBL genes including: *bla*_CTX-M-15_, and *bla*_SHV-12_. Moreover, ARGs against aminoglycosides (*aac(6’)-Ib-cr)*, trimethoprim-sulfamethoxazole (*dfrA-17, dfrA-19, dfrA-7, and dfrA-14*), phenicols (*catB3*), and sulfonamides (*sul1* and *sul2*) were commonly observed. Genomic analysis identified the *E. coli* isolates as ST410, which is associated with the pandemic extraintestinal pathogenic group (ExPEC). It belonged to the O96:H serotype, and its phylogroup was categorized as phylogroup C. Among the numerous virulence genes detected in this isolate, notable ExPEC-associated virulence genes such as *papC, afra/dra*, and *iutA* were present. The *K. pneumoniae* isolate was identified as ST337, with the capsular locus KL109, and the O2v2 O-lipopolysaccharides type. Similarly, *K. michiganensis,* when analyzed using the Kaptive/Holt lab database, was found to carry the capsular locus KL107, and the O1v1 O-lipopolysaccharides type. The *E. cloacae* were polyclonal including ST114 (n = 3), ST184 (n = 3), ST254 (n = 1) and *E. mori* ST2791 (n = 1). ST114, ST184 and ST254 were commonly associated with nosocomial infection.

**Table 3:** Species identity, resistome profile, and plasmid replicon types in isolates carrying the mcr-9-genes.

Isolate ID	Species	ST	Antimicrobial resistance genes	Plasmid/replicon types
p165e	*E. coli*	410	mcr-9, *bla*_CTX-M-15_, aac *(6’)-Ib-cr, aac (6’) IIc*, aph (3’)-Ia, *aadA5*, *bla*_SHV-12_, *bla*_OXA-1_, *bla*_CMY-2_, tet(B), dfrA17, and mph(A)	*IncFII, IncHI2, IncHI2A*
p004kp	*K. pneumoniae*	337	mcr-9, *bla*_TEM-1B,_ *aac (6’) IIc, bla*_SHV-12_, _−40_, _−56_, _−79,_ −_89_, *sul1, sul2, fosA, oqxA, and oqxB,*	*IncFII, IncHI2*, *IncHI2A*
s082km	*K. michiganensis*	na	mcr-9, *bla*_OXY-1–2_, *bla*_TEM-1B_, *aac (6’) Ib-cr, aac (6’) IIc, aac (6’) Ib3, sul1, sul2.*	*IncHI2*
m016ECL	*E. mori*	2197	mcr-9, *bla*_CTX-M-15_, bla_SHV-12_, *bla*_TEM-1B_, *aph (3”)-Ib, aac (6’)-IIc, aph (6)-Id, dfrA19, sul1, sul2, qnrB2,*	*IncHI2, IncHI2A*
m481ECL	*E. cloacae*	114	mcr-9, *bla*_CTX-M-15_, bla_OXA-1_, *bla*_SHV-12_, *bla*_ACT-16_, *aac (6’)-IIc, aac (6’) Ib-cr, aph (3’)-Ia, aph (6)-Id, dfrA19, sul1, sul2, catB3, ARR-3*	*IncHI2, IncHI2A, IncX3*
s164ECL	*E. cloacae*	254	mcr-9, *bla*_SHV-12_, bla_TEM-1B_, *aac (6’)-IIc, aph (6)-Id, dfrA19, sul1, sul2, qnrB2*	*IncHI2, IncHI2A, IncX5*
s202ECL	*E. cloacae*	114	mcr-9, *bla*_CTX-M-15_, bla_OXA-1_, *bla*_SHV-12_, aac (6’)-Ib-cr, aac (6’)-IIc, aph (3”)-Ib, aph (6)-Id, dfrA19, sul1, sul2, qnrB2, catB3, ARR-3	*IncHI2, IncHI2A, IncX3*
s249ECL	*E. cloacae*	114	mcr-9, *bla*_CTX-M-15_, *bla*_SHV-12_, *aac (6’)-Ib-cr, aac (6’)-IIc, aph (3’)-Ia, aph (6)-Id, dfrA19, sul1, sul2,*	*IncHI2, IncHI2A, IncX3*
s257ECL	*E. cloacae*	184	mcr-9, *bla*_CTX-M-15_, *bla*_OXA-1_, *bla*_SHV-12_, bla_TEM-1B_, *aac (6’)-Ib-cr, aph (3’‘)-Ib, dfrA14, dfrA7, sul1, sul2, tet(A), qnrB1*	*IncHI2, IncHI2A IncFIB(pHCM2), IncM1*
s284ECL	*E. cloacae*	184	mcr-9, *bla*_CTX-M-15_, *bla*_OXA-1_, *bla*_SHV-12_, *bla*_TEM-1B_, *aac (6’)-Ib-cr, aph (3’‘)-Ib, aac (6’)-IIc, dfrA14, dfrA7, sul1, sul2, tet(A), qnrB1*	*IncHI2, IncHI2, A IncFIIB*
s304ECL	*E. cloacae*	184	mcr-9, *bla*_CTX-M-15_, *bla*_OXA-1_, *bla*_SHV-12_, *bla*_TEM-1B_, *aac (6’)-Ib-cr, aph (3’‘)-Ib, aac (6’)-IIc, dfrA14, dfrA7, sul1/sul2, tet(A), qnrB1, qnrS1*	*IncHI2, IncHI2A, IncFIB, IncFIIB, IncHI1B, IncM1*

**Key:** ST- sequence type, na- not applicable, novel- Sequence type not assigned.

### Genetic structure and content of plasmids encoding *mcr-9*

**Fig 1:**
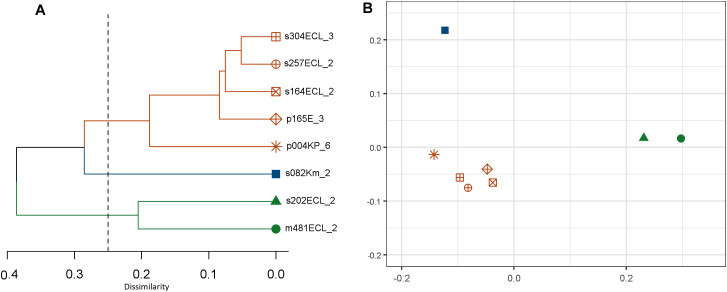
Clustering recovered plasmids that contain mcr-9 using Bray-Curtis measure of their gene-content. A) a dendrogram showing cluster of plasmids with 25% dissimilarity in gene content. B) a PCoA plot showing the overall dissimilarity plasmids in their gene-content.

Multiple plasmid replicon types were identified in all the isolates which included *IncHI2*, *IncHI2A* and *IncX3*. The *IncHI2* replicon was most prevalent found in all isolates, followed by *IncHI2A* as the second most prevalent type details are shown in Table 3. All the plasmids encoding the *mcr-9* had similar replicon profiles, and the mcr-9 genes were located on *IncHI2* replicon types. The sequenced plasmids carrying the mcr-9 had different size. The backbones of the plasmids were mapped to previously sequenced plasmids from the NCBI database (S1-S8_File.pdf in S1 File). However, the eight characterized plasmids formed three clusters regarding gene content (Fig 1), highlighting their different evolutionary path and accessibility of probable co-selection of *mcr-9* with other functions.

**Fig 2:**
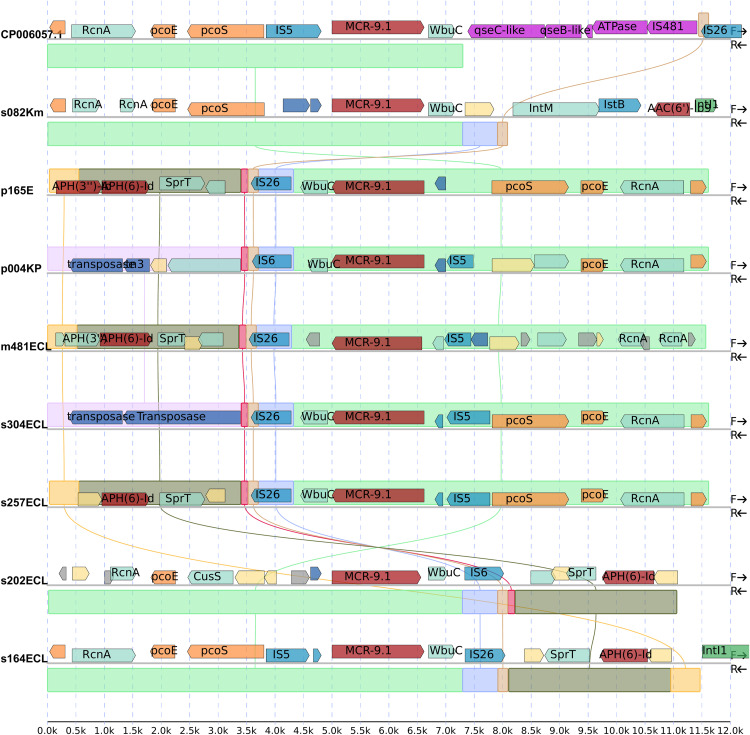
The immediate genetic context of *mcr-9* encoding plasmids compared to standard reference plasmid from Salmonella enterica (CP006057.1). Aligned regions are shown by colored rectangles and the annotated ORFs are plotted with arrows in forward strand. A genetic context upstream of *mcr-9*–*wbuC* complex are almost intact in all studied plasmids (the light green block). However, the downstream were quite dissimilar.

Moreover, Fig 2 shows that the plasmids contain similar genetic contexts upstream of the *mcr-9,* which present in all the recovered genetic contexts (the green rectangle in Fig 2). Nonetheless, the variations observed in the downstream region underscore the occurrence of separate transpositional events involving the *mcr-9–wbuC* complex within distinct plasmids or their subsequent downstream regions, as illustrated by the rectangles with different colors in Fi 2. It is noteworthy that all the retrieved genetic contexts lack the specific genetic elements that encompass *qseC*- and *qseB*-like genes, as indicated in pink within the reference genome in Fig 2. However, the remnant of IS*26* identified beyond *qseC*- and *qseB*-like genes region matches with the same gene downstream of *mcr-9*–*wbuC* complex, suggesting that a separate transposition event might have inserted the missing region in the *Salmonella enterica* genome (CP006057.1) which can express *mcr-9*.

## Discussion

The current study presents the silent *mcr* −9 gene and its genetic context in clinical isolates of *Enterobacteriaceae* from Ethiopia. The spread of *mcr*-genes through several variants *mcr-1* and *mcr-10* over wider geographical areas has become a global threat. We detected the *mcr-9* genes in four different species of ESBL-producing clinical isolates of *Enterobacteriaceae* when the use of colistin was not documented in the country. However, Ethiopia has recently included colistin in the national list of essential medicines for use in clinical setting (http://efmhaca.hcmisonline.org/wp-content/uploads/2020/12/EML-sixth-edition.pdf).

Despite encoding *mcr-9*, isolates in the current study were susceptible for colistin. The clinical use of colistin may apply selective pressure that induces expression of the *mcr-9* gene. The core genetic context of *mcr-9* and *wbuC*, was the same in most of the plasmids in this study. But as compared to previous studies, in the genetic context of *mcr-9* (the flanking regions) *qseC*-like and *qseB*-like genes which are believed to function as regulatory genes [8,21] are missing. However, it worth mentioning that a study from United States reported *S. enterica* and *E. coli* strains with *qseC* and *qseB* genes susceptible for colistin (MIC ≤ 1) [22]. It could suggest that *mcr-9* gene is silent in these contexts, and most likely the reason that all the isolates were susceptible to colistin with MIC less than 0.5 mg/L is due to the absence of this upstream region regulatory gene component.

Moreover, the presence of multiple resistance genes in addition to the *mcr-9* increases risk of co-selection. Acquisition of the *qseC/qseB* [8] or other undetermined mechanisms (even in the absence of *qseC/*qseB) like the *arnBCADTEF* gene cassette which are genospecies specific mechanisms of resistance to colistin might interact with *mcr-9* and enhance the selection and dissemination of the gene [23]. It has also been demonstrated that in a hybrid plasmid *IncHI2/IncHI2A* encoding *bla*_NDM-1_, *mcr-9* has shown increased expression and consequently phenotypic resistance following induction with colistin [24]. Thus, activation of the gene could occur either through mobilization of the gene by MGEs and consequently its integration into a fully functional genetic contexts or through the acquisition of novel regulatory mechanisms driven by mutations or horizonatal genetic transfer. Additionally, the coexistence of ARGs against other antibiotic classes facilitates the dissemination of *mcr-9* across species and lineages via co-selection mechanisms [25].

The spread of a silent *mcr-9* gene extends beyond the findings of this study. For example, a study from Ethiopia [26] identified two cases of *mcr-9* in isolates obtained from two geographically distant tertiary hospitals. One isolate was an unclassified *Salmonella* species, and the other was *Klebsiella pneumoniae*, both associated with bloodstream infections. Notably, similar to the findings in the current study, both isolates remained susceptible to colistin. These findings highlight the potential for wider dissemination of *mcr-9* and emphasize the need for molecular surveillance.

There are some reports of *mcr-9* from human clinical samples in Africa; mainly one from South Africa [27] and the other from Egypt [28]. Despite the limited studies among clinical samples in the continent, *mcr* genes were reported from several other ecosystems including human carriers, animals, environment, and food products [29]. Furthermore, the limited diagnostic capacity might have obscured the actual prevalence of colistin resistance and *mcr* genes. On the other hand, the lack of strict control of antimicrobial usage, and lack of stringent infection prevention and control strategies can catalyze the spread of colistin resistance and/or spread of the *mcr-9* genes and may continue to pose a challenge in both human and animal health.

The transmission of *mcr* genes is commonly through polyclonal strains, and mostly one or two species were reported from different studies. In the current study, the detection of *mcr-9* from four different *Enterobacteriaceae* species, and one of them *E. cloacae* belonging to different sequence types, may indicate that there is an ongoing silent spread of the gene. Furthermore, in the apparent global perspective, *E. cloacae* complex seem more prone to harboring and spreading the *mcr-9* mediated colistin resistance and also the silent spread of the gene [30–32].

The mechanism of spread for *mcr-9* genes is mediated by plasmids, all the *mcr-9* genes were located on either *IncHI2* or *IncHI2A* replicon types. The genetic content and location of the *mcr-9* gene differed between these plasmids. Clustering analysis showed that the plasmids in this study have some differences regarding their genetic content. Similar findings were reported from previous studies that *IncHI2*/*IncHI2A* were carriers of the *mcr-9* genes [8,28,33]. Moreover, in the presence of multiple plasmid replicon types in all the isolates, *mcr-9* can be transposed to other plasmids that may encode transcription factors necessary for expressing phenotypic resistance.

The *mcr-9* gene carried on the super-plasmids *IncHI2* can be co-selected with other ARGs (like the extended spectrum cephalosporins, aminoglycosides, fluoroquinolones, trimethoprim, and sulfonamides), and mobilized with a range of mobile genetic elements. A global review of literature regarding *mcr-9* isolates and plasmids showed that the *IncHI2* plasmids were responsible for the transmission of *mcr-9* in several countries. Apparently, the silent spread of *mcr-9* without phenotypic resistance seems to have followed similar trend as in other parts of the world [29]. In Africa, the lack of targeted epidemiological data may have obscured the extent of silent spread of mcr-9 and colistin resistance in general.

Despite mcr-9 being first described in 2019 and availability of sporadic reports from different regions; retrospective analysis of genomic data collected earlier offers valuable insights into the emergence of mcr-9 and its epidemiology, particularly in resource-limited settings such as Africa. Many bacterial isolates sequenced prior to the identification of mcr-9 were not screened for this gene, resulting in a critical knowledge gap. By re-examining these historical datasets using current bioinformatics tools and updated resistance gene databases, we identified previously unrecognized occurrences of mcr-9 in higher number than previously reported from a human source and at a a clinical settings in Africa, thereby establishing a more accurate timeline and geographical distribution of genetic determinant in Ethiopia. This finding is important in understanding evolutionary origins, and early reservoirs of mcr-9, which can inform a more effective surveillance and control strategies. In Africa, where active genomic surveillance remains limited, leveraging existing datasets enhance epidemiological understanding as complemenatry approach to active surveillance. Integrating such historical genomic epidemiology data into global antimicrobial resistance knowledge pool is therefore not only beneficial but also addresses the underreporting and delayed recognition of emerging resistance genes like mcr-9 across the continent.

## Conclusion

The detection of the *mcr-9* gene in multiple species of clinical isolates—despite their susceptibility to colistin—carried on conjugative plasmids with similar replicon types but diverse genetic content and an array of MGEs, is highly concerning. Colistin has not been included as national list of drugs in Ehtiopia in 2016 when these isolates were collected. The clinical use of colistin should be carefully regulated in settings where further mobilization and co-selection of silent *mcr-9* could potentially lead to the emergence of phenotypic resistance. However, susceptibility of these strains to carbapenems, and beta-lactam/beta-lactam inhibitor combinations is important clinical information for current consideration.

## Supporting information

S1 File Sequence alignment of 40 IncHI2 *mcr-9* encoding plasmids and their genetic content compared to plasmid from 8 of the isolates where we detected mcr-9. The outer most structure in each of the eight figures below shows the genetic structure of plasmids from each of the isolates from the current study, and the gaps indicated the absence a gene.
(PDF)

## References

[1] Acman M, Wang R, van Dorp L, Shaw LP, Wang Q, Luhmann N, et al. Role of mobile genetic elements in the global dissemination of the carbapenem resistance gene blaNDM. Nat Commun. 2022;13(1):1131. doi: 10.1038/s41467-022-28819-2 35241674 PMC8894482

[2] Frost LS, Leplae R, Summers AO, Toussaint A. Mobile genetic elements: the agents of open source evolution. Nat Rev Microbiol. 2005;3(9):722–32. doi: 10.1038/nrmicro1235 16138100

[3] Haudiquet M, Moura de Sousa J, Touchon M. Selfish, promiscuous, and sometimes useful: how mobile genetic elements drive horizontal gene transfer in microbial populations. EvoEcoRxiv. 2021;:1–21. doi: 10.1098/rstb.2015.0615 PMC9393566 35989606

[4] WHO Advisory Group. WHO list of critically important antimicrobials (CIA) 2019. https://www.who.int/groups/advisory-group-on-the-who-list-of-critically-important-antimicrobials. Accessed 2025 October 29

[5] Chatzidimitriou M, Kavvada A, Kavvadas D, Kyriazidi MA, Meletis G, Chatzopoulou F, et al. mcr Genes Conferring Colistin Resistance in Enterobacterales; a Five Year Overview. Acta Med Acad. 2021;50(3):365–71. doi: 10.5644/ama2006-124.355 35164512

[6] Mmatli M, Mbelle NM, Osei Sekyere J. Global epidemiology, genetic environment, risk factors and therapeutic prospects of mcr genes: A current and emerging update. Front Cell Infect Microbiol. 2022;12:941358. doi: 10.3389/fcimb.2022.941358 36093193 PMC9462459

[7] Carroll LM, Gaballa A, Guldimann C. Identification of novel mobilized colistin resistance gene mcr-9 in a multidrug-resistant, colistin-susceptible Salmonella enterica serotype Typhimurium. J Antimicrobial Chemotherapy. 2019;10:1–6. 10.1128/mBio.00853-19 PMC6509194 31064835

[8] Kieffer N, Royer G, Decousser J-W, Bourrel A-S, Palmieri M, Ortiz De La Rosa J-M, et al. mcr-9, an Inducible Gene Encoding an Acquired Phosphoethanolamine Transferase in Escherichia coli, and Its Origin. Antimicrob Agents Chemother. 2019;63(9):e00965-19. doi: 10.1128/AAC.00965-19 31209009 PMC6709461

[9] Dadashi M, Sameni F, Bostanshirin N, Yaslianifard S, Khosravi-Dehaghi N, Nasiri MJ, et al. Global prevalence and molecular epidemiology of mcr-mediated colistin resistance in Escherichia coli clinical isolates: a systematic review. J Glob Antimicrob Resist. 2022;29:444–61. doi: 10.1016/j.jgar.2021.10.022 34788692

[10] Elbediwi M, Li Y, Paudyal N, Pan H, Li X, Xie S, et al. Global burden of colistin-resistant bacteria: mobilized colistin resistance genes study (1980-2018). Microorganisms. 2019;7(10):461. doi: 10.3390/microorganisms7100461 31623244 PMC6843232

[11] Mariani B, Corbella M, Merla C, Tallarita M, Piralla A, Girello A, et al. Bloodstream infections caused by Escherichia coli carrying mcr-1 gene in hospitalized patients in northern Italy from 2012 to 2018. Infection. 2020;48(2):223–30. doi: 10.1007/s15010-019-01377-4 31758437

[12] Nordmann P, Lienhard R, Kieffer N, Clerc O, Poirel L. Plasmid-Mediated Colistin-Resistant Escherichia coli in Bacteremia in Switzerland. Clin Infect Dis. 2016;62(10):1322–3. doi: 10.1093/cid/ciw124 26936673

[13] Huang H, Dong N, Shu L, Lu J, Sun Q, Chan EW-C, et al. Colistin-resistance gene mcr in clinical carbapenem-resistant Enterobacteriaceae strains in China, 2014-2019. Emerg Microbes Infect. 2020;9(1):237–45. doi: 10.1080/22221751.2020.1717380 31996107 PMC7034111

[14] Velasco JMS, Valderama MTG, Margulieux KR, Diones PCS, Reyes AMB, Leonardia SG, et al. First report of the mcr-1 colistin resistance gene identified in two Escherichia coli isolates from clinical samples, Philippines, 2018. J Glob Antimicrob Resist. 2020;21:291–3. doi: 10.1016/j.jgar.2019.12.018 31927060

[15] Bitar I, Papagiannitsis CC, Kraftova L, Chudejova K, Mattioni Marchetti V, Hrabak J. Detection of five mcr-9-carrying enterobacterales isolates in four czech hospitals. mSphere. 2020;5(6):e01008-20. doi: 10.1128/mSphere.01008-20 33298573 PMC7729258

[16] Song K, Jin L, Cai M, Wang Q, Wu X, Wang S, et al. Decoding the origins, spread, and global risks of mcr-9 gene. EBioMedicine. 2024;108:105326. doi: 10.1016/j.ebiom.2024.105326 39260038 PMC11416231

[17] Sewunet T, Asrat D, Woldeamanuel Y, Ny S, Westerlund F, Aseffa A, et al. Polyclonal spread of blaCTX-M-15 through high-risk clones of Escherichia coli at a tertiary hospital in Ethiopia. J Glob Antimicrob Resist. 2022;29:405–12. doi: 10.1016/j.jgar.2021.09.017 34775133

[18] Sewunet T, Asrat D, Woldeamanuel Y, Ny S, Westerlund F, Aseffa A, et al. High prevalence of blaCTX-M-15 and nosocomial transmission of hypervirulent epidemic clones of Klebsiella pneumoniae at a tertiary hospital in Ethiopia. JAC Antimicrob Resist. 2021;3(1):dlab001. doi: 10.1093/jacamr/dlab001 34223080 PMC8210115

[19] K F, Galore T. A wrapper tool around Cutadapt and FastQC to consistently apply quality and adapter trimming to FastQ files. 2015;516(517).

[20] Camacho C, Coulouris G, Avagyan V, Ma N, Papadopoulos J, Bealer K, et al. BLAST+: architecture and applications. BMC Bioinformatics. 2009;10:421. doi: 10.1186/1471-2105-10-421 20003500 PMC2803857

[21] Ribeiro TG, Izdebski R, Urbanowicz P, Carmeli Y, Gniadkowski M, Peixe L. Citrobacter telavivum sp. nov. with chromosomal mcr-9 from hospitalized patients. Eur J Clin Microbiol Infect Dis. 2021;40(1):123–31. doi: 10.1007/s10096-020-04003-6 32808110

[22] Tyson GH, Li C, Hsu C-H, Ayers S, Borenstein S, Mukherjee S, et al. The mcr-9 gene of salmonella and escherichia coli is not associated with colistin resistance in the United States. Antimicrob Agents Chemother. 2020;64(8):e00573-20. doi: 10.1128/AAC.00573-20 32513803 PMC7526823

[23] Doijad SP, Gisch N, Frantz R, Kumbhar BV, Falgenhauer J, Imirzalioglu C, et al. Resolving colistin resistance and heteroresistance in Enterobacter species. Nat Commun. 2023;14(1):140. doi: 10.1038/s41467-022-35717-0 36627272 PMC9832134

[24] Liu Z, Hang X, Xiao X, Chu W, Li X, Liu Y, et al. Co-occurrence of blaNDM-1 and mcr-9 in a Conjugative IncHI2/HI2A Plasmid From a Bloodstream Infection-Causing Carbapenem-Resistant Klebsiella pneumoniae. Front Microbiol. 2021;12:756201. doi: 10.3389/fmicb.2021.756201 34956120 PMC8701513

[25] Lee KY, Hopkins JD, Syvanen M. Direct involvement of IS26 in an antibiotic resistance operon. 1990;172. 10.1128/jb.172.6.3229-3236.1990 PMC209129 2160941

[26] Legese MH, Asrat D, Mihret A, Hasan B, Mekasha A, Aseffa A, et al. Genomic epidemiology of carbapenemase-producing and colistin-resistant enterobacteriaceae among sepsis patients in Ethiopia: a whole-genome analysis. Antimicrob Agents Chemother. 2022;66(8):e0053422. doi: 10.1128/aac.00534-22 35876577 PMC9380574

[27] Osei Sekyere J, Reta MA. Genomic and resistance epidemiology of gram-negative bacteria in Africa: a systematic review and phylogenomic analyses from a one health perspective. mSystems. 2020;5(6):e00897-20. doi: 10.1128/mSystems.00897-20 33234606 PMC7687029

[28] Sadek M, Nariya H, Shimamoto T. First genomic characterization of bla VIM-1 and mcr-9- coharbouring Enterobacter hormaechei. 2020;:1–7. 10.3390/pathogens9090687 PMC7558541 32842587

[29] Anyanwu MU, Okpala COR, Chah KF, Shoyinka VS. Prevalence and Traits of Mobile Colistin Resistance Gene Harbouring Isolates from Different Ecosystems in Africa. Biomed Res Int. 2021;2021:6630379. doi: 10.1155/2021/6630379 33553426 PMC7847340

[30] Li Y, Dai X, Zeng J, Gao Y, Zhang Z, Zhang L. Characterization of the global distribution and diversified plasmid reservoirs of the colistin resistance gene mcr-9. Sci Rep. 2020;10(1):8113. doi: 10.1038/s41598-020-65106-w 32415232 PMC7229202

[31] Lin M, Yang Y, Yang Y, Chen G, He R, Wu Y, et al. Co-Occurrence of mcr-9 and blaNDM-1 in Enterobacter cloacae Isolated from a Patient with Bloodstream Infection. Infect Drug Resist. 2020;13:1397–402. doi: 10.2147/IDR.S248342 32494170 PMC7229791

[32] Babiker A, Bower C, Lutgring JD. Clinical and genomic epidemiology of mcr-9 -carrying carbapenem-resistant Enterobacterales isolates in metropolitan. 2017. 10.1128/spectrum.02522-21 PMC9431279 35856667

[33] Diaconu EL, Alba P, Feltrin F, Di Matteo P, Iurescia M, Chelli E, et al. Emergence of IncHI2 plasmids with mobilized colistin resistance (mcr)-9 gene in ESBL-producing, multidrug-resistant salmonella typhimurium and Its monophasic variant ST34 from food-producing animals in italy. Front Microbiol. 2021;12:705230. doi: 10.3389/fmicb.2021.705230 34335538 PMC8322855

