# Supplementary material for: Silent spread of mcr-9 in ESBL-producing Enterobacteriaceae clinical isolates, Jimma, Ethiopia
Source: PLoS One. 2025 Nov 18;20(11):e0336440. doi: 10.1371/journal.pone.0336440 (PMC12626256; doi:10.1371/journal.pone.0336440)
Supplement: S1 File — The outer most structure in each of the eight figures below shows the genetic structure of plasmids from each of the isolates from the current study, and the gaps indicated the absence a gene. (PDF) [file pone.0336440.s001.pdf]

Supplementary Figures (S1-S8): Showing sequence alignment of 40 *IncHI2* mcr-9 encoding plasmids and their genetic content compared to plasmid from 8 of the isolates where we detected *mcr-9*. The outer most structure in each of the eight figures below shows the genetic structure of plasmids from each of the isolates from the current study, and the gaps indicated the absence a gene.

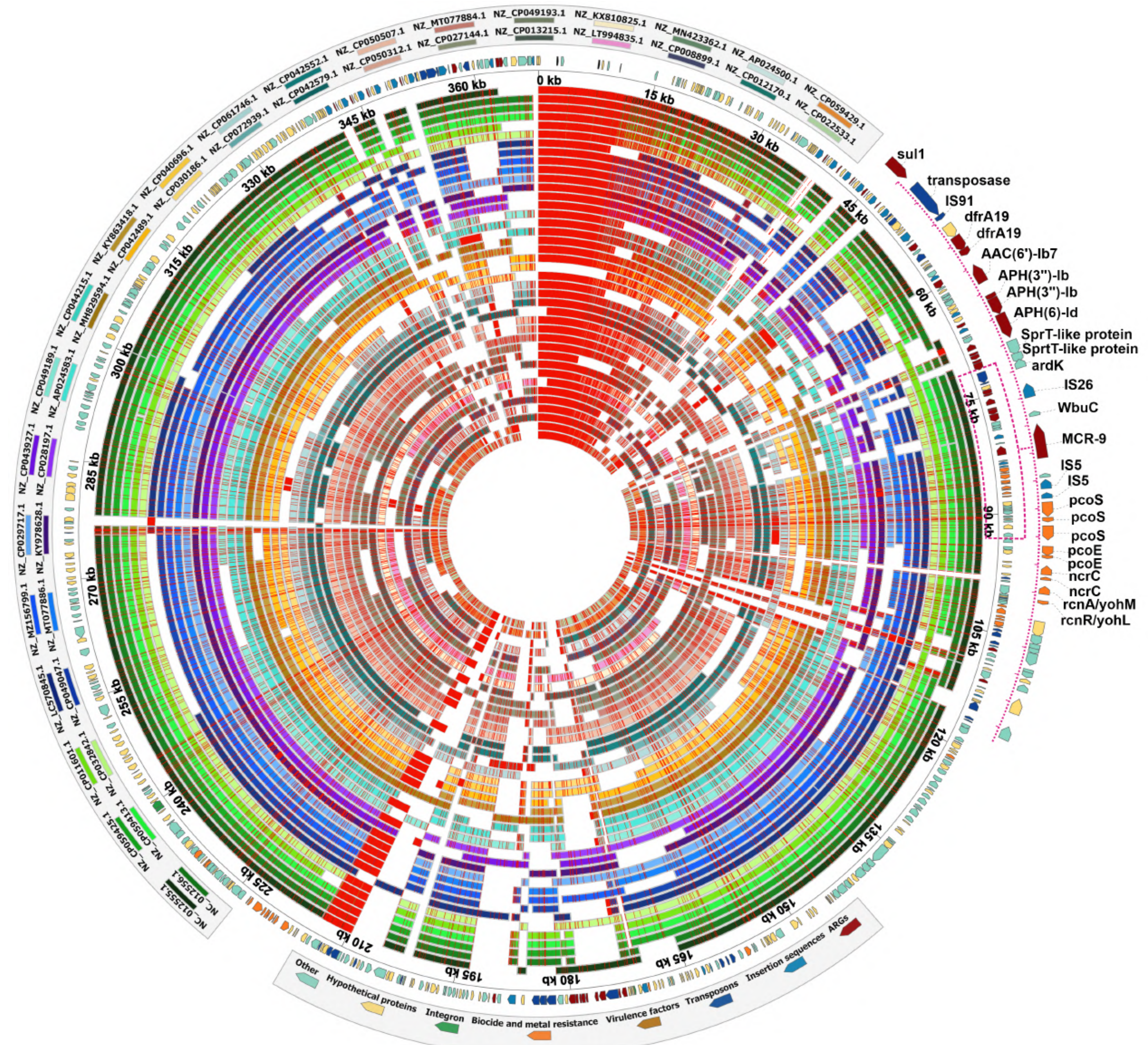

S1: Genome of a plasmid labeled m481ECL from *Enterobacter cloacae* isolated at the medical ward is mapped against 40 plasmids from NCBI.

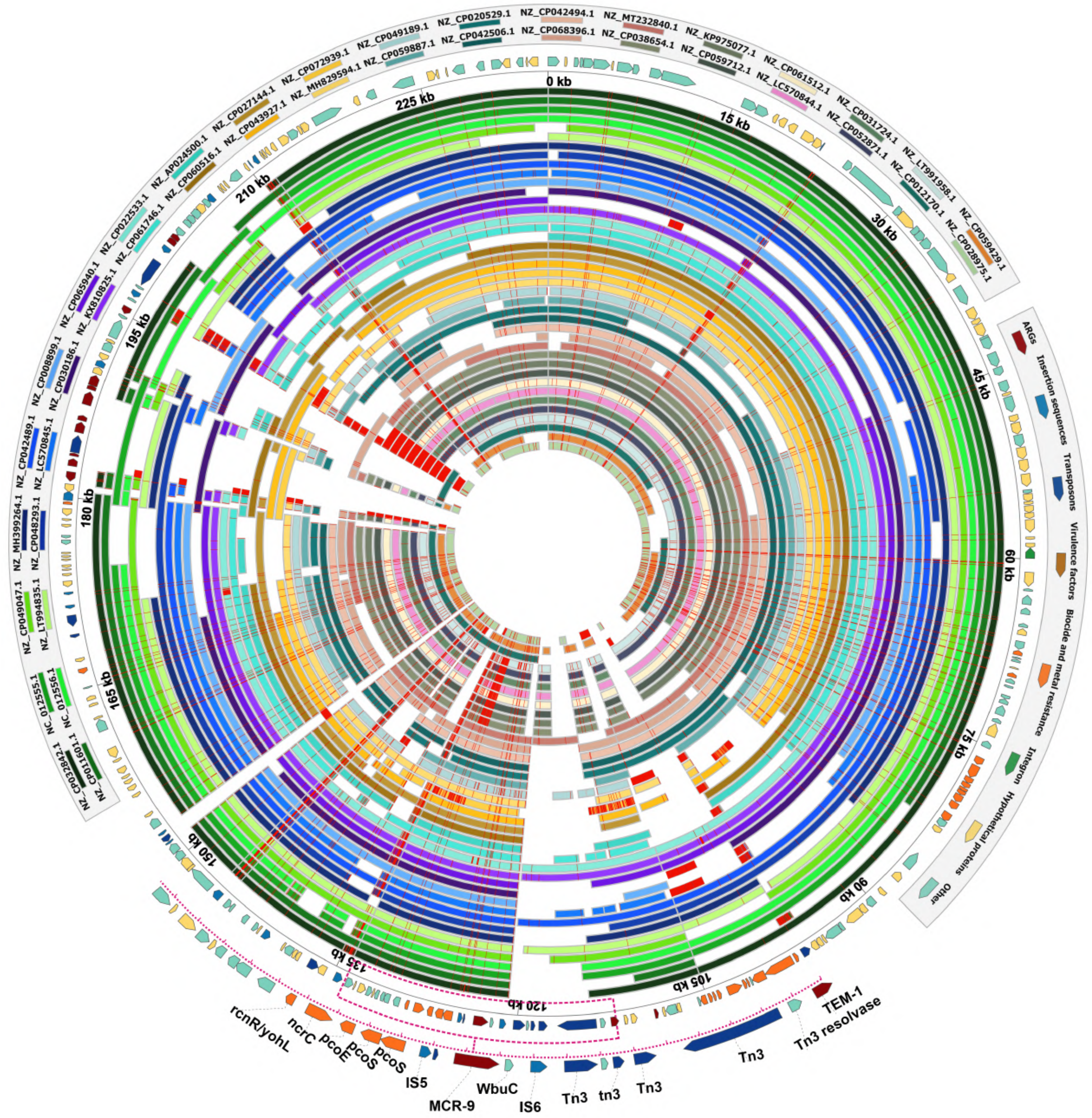

S2: Genome of a plasmid labelled p004KP from *K. pneumoniae* isolated at the pediatric ward is mapped against 40 plasmids from NCBI.

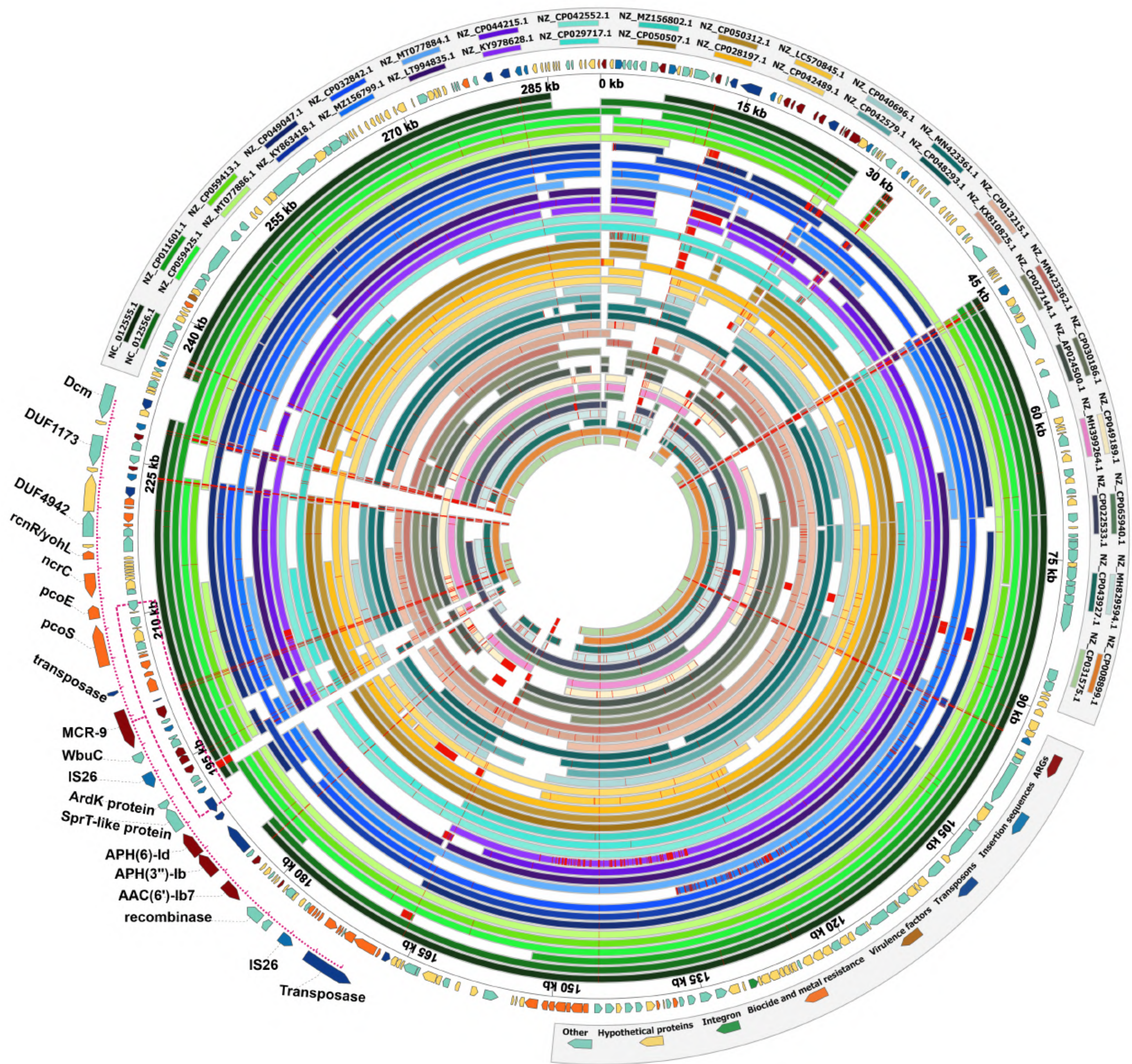

S3: Genome of a plasmid labeled p165E from *Escherichia coli* isolated at the pediatric ward is mapped against 40 plasmids from NCBI.

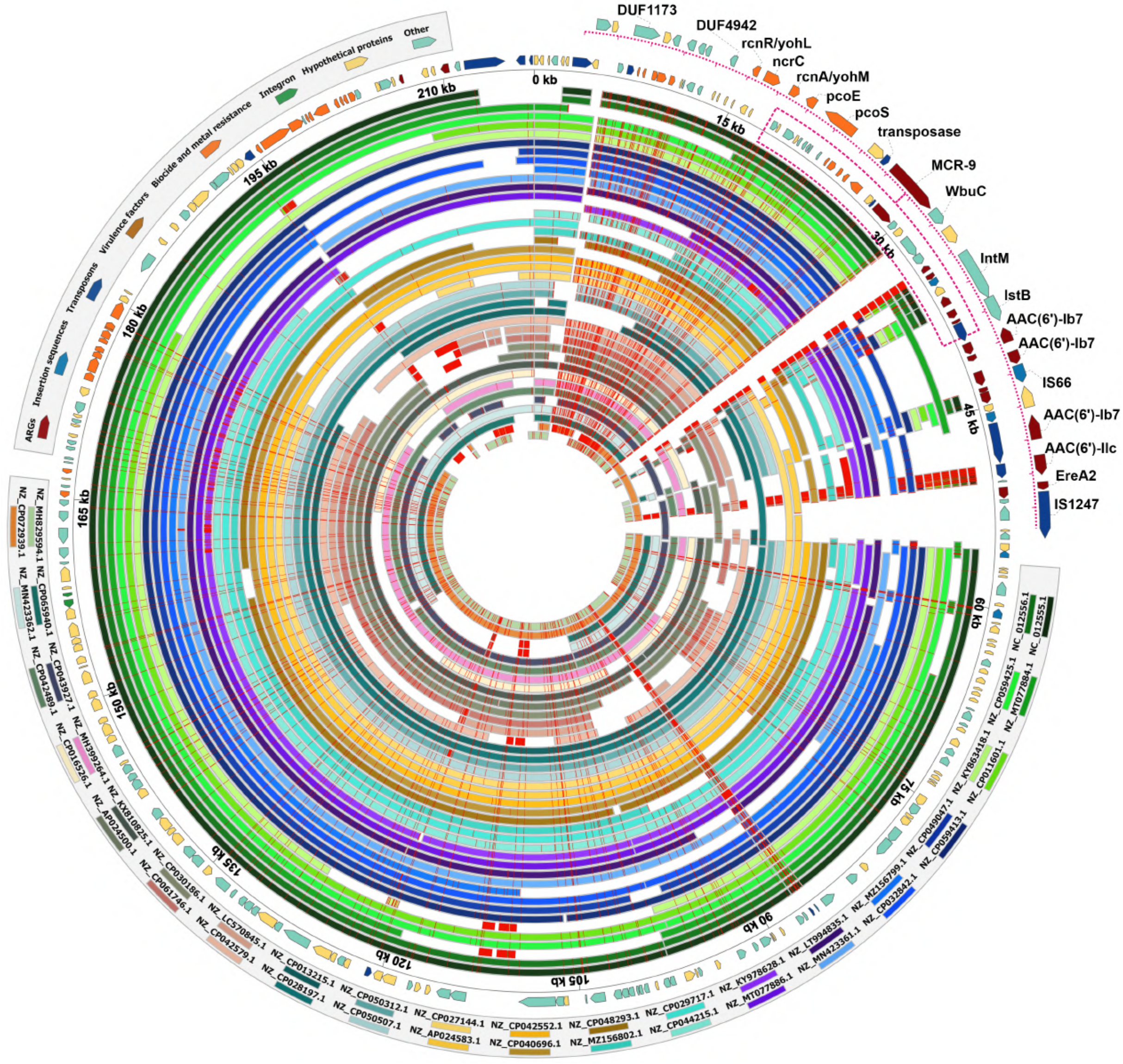

S4: Genome of a plasmid labeled s082KM from *K. michiganensis* isolated at the surgical ward is mapped against 40 plasmids from NCBI.

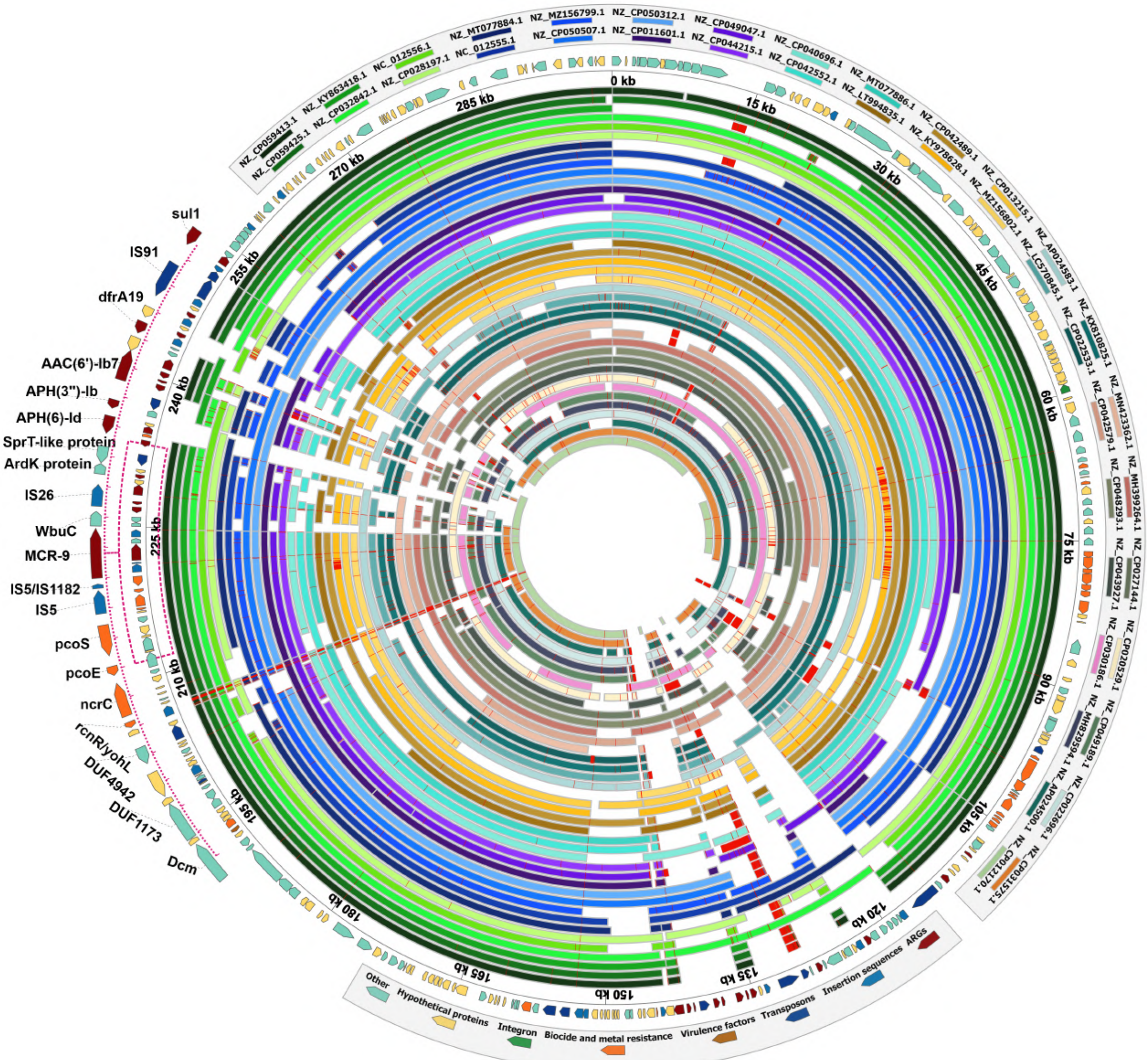

S5: Genome of a plasmid labeled s164ECLl from *Enterobacter cloacae* isolated at the surgical ward is mapped against 40 plasmids from NCBI.

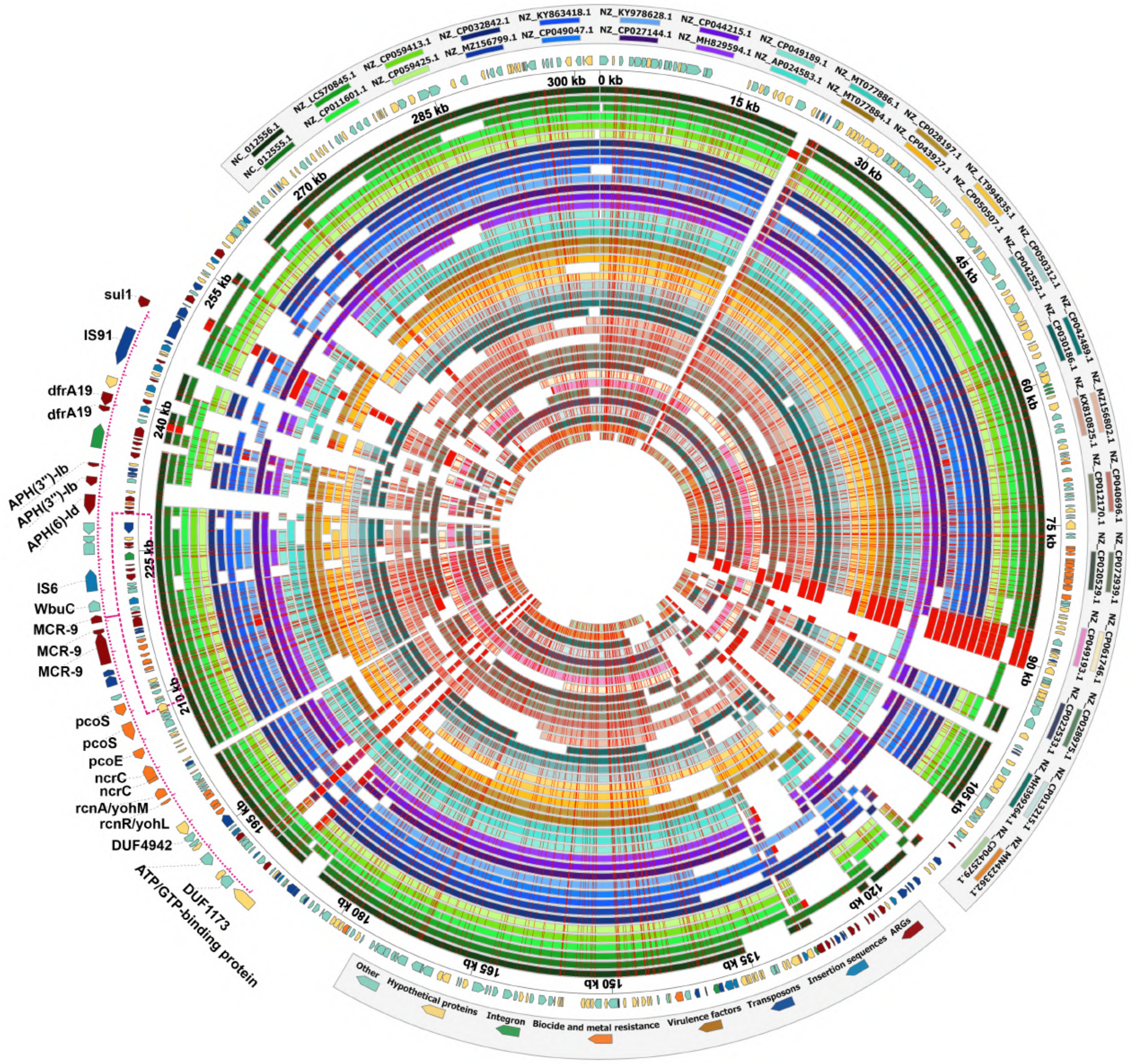

S6: s202ecl Genome of a plasmid labeled s202ECL from *Enterobacter cloacae* isolated at the surgical ward is mapped against 40 plasmids from NCBI.

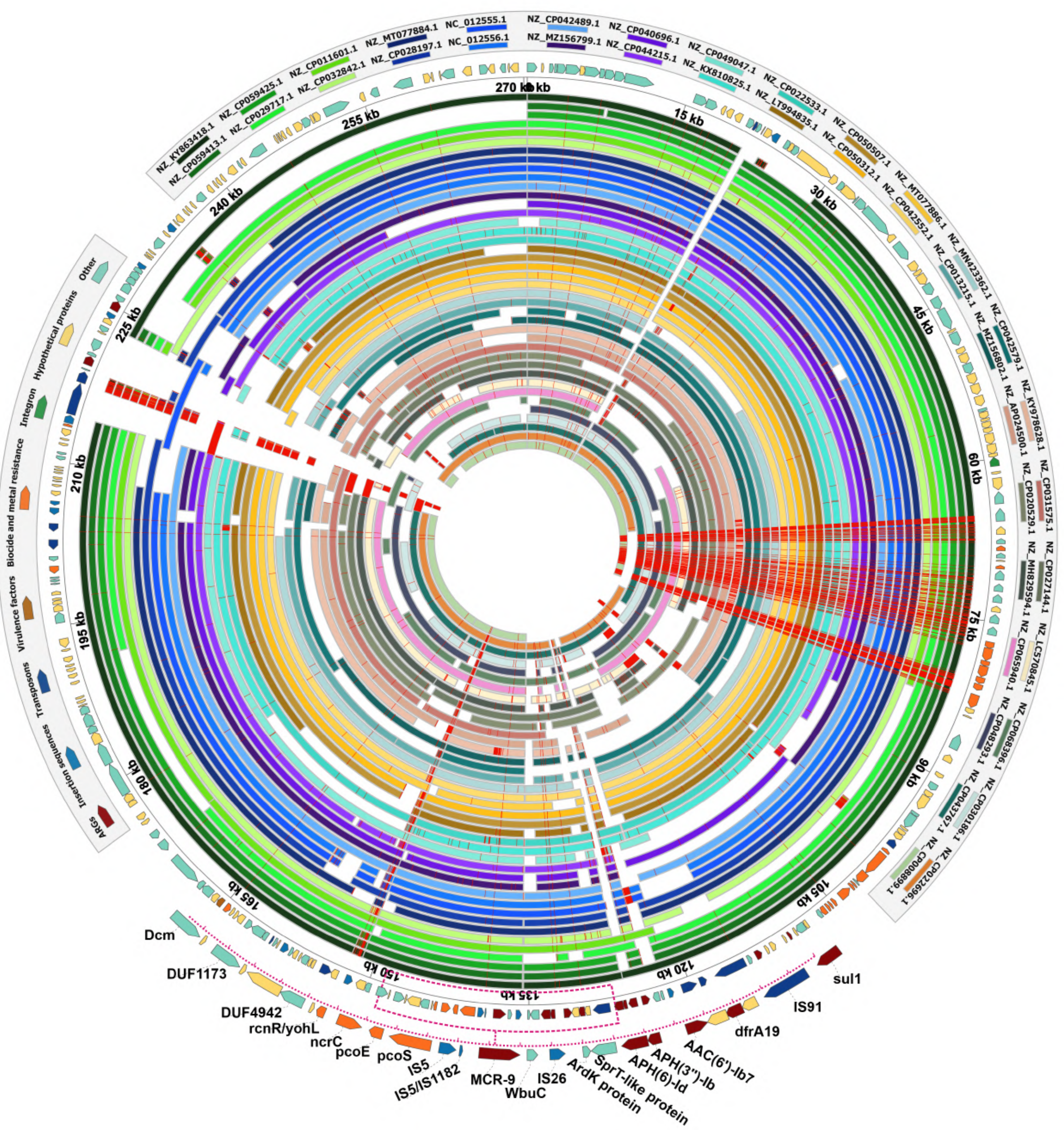

S7: Genome of a plasmid labeled s257ECL from Enterobacter *cloacae* isolated at the surgical ward is mapped against 40 plasmids from NCBI.

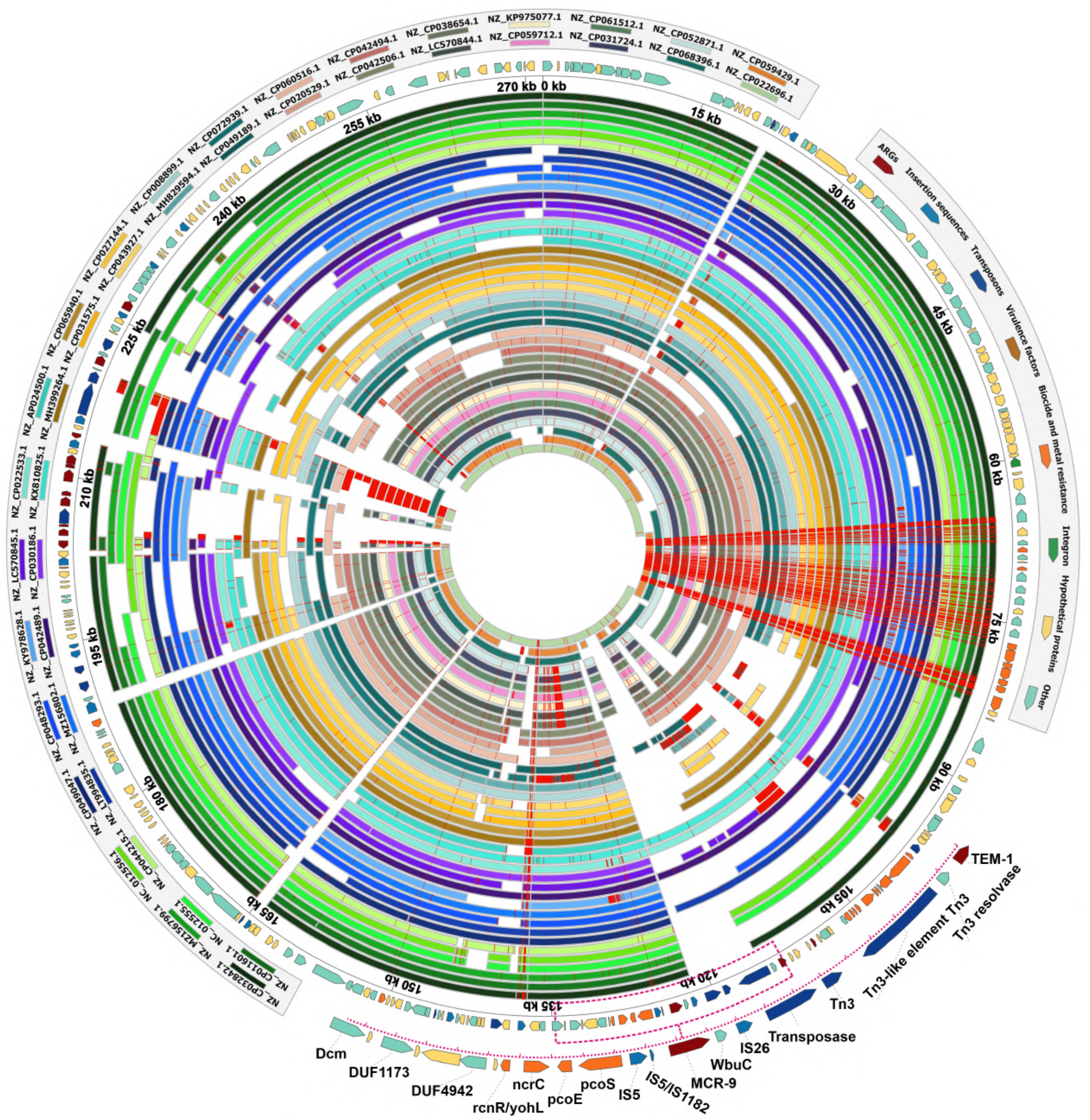

S8: Genome of a plasmid labeled s304ECL from Enterobacter *cloacae* isolated at the surgical ward is mapped against 40 plasmids from NCBI.
